# Intravitreal Therapy Against the Complement Factor C5 Prevents Retinal Degeneration in an Experimental Autoimmune Glaucoma Model

**DOI:** 10.3389/fphar.2019.01381

**Published:** 2019-12-02

**Authors:** Sabrina Reinehr, Sara C. Gomes, Caroline J. Gassel, M. Ali Asaad, Gesa Stute, Marc Schargus, H. Burkhard Dick, Stephanie C. Joachim

**Affiliations:** ^1^Experimental Eye Research Institute, University Eye Hospital, Ruhr-University Bochum, Bochum, Germany; ^2^Department of Ophthalmology, University Hospital Duesseldorf, Heinrich-Heine-University Duesseldorf, Duesseldorf, Germany

**Keywords:** glaucoma, complement, complement inhibition, membrane attack complex, optical coherence tomography

## Abstract

In glaucoma, studies revealed an involvement of the complement system. In an experimental autoimmune glaucoma model, immunization with an optic nerve homogenate antigen (ONA) led to retinal ganglion cell (RGC) loss, while intraocular pressure (IOP) remained unchanged. Here, we investigated the therapeutic effect of a complement system inhibition in this model. Hence, rats were immunized with ONA and compared to controls. In one eye of the ONA animals, an antibody against complement factor C5 was intravitreally injected (15 μmol: ONA+C5-I or 25 μmol: ONA+C5-II) before immunization and then every two weeks. IOP was measured weekly. After 6 weeks, spectral-domain optical coherence tomographies (SD-OCT), electroretinograms (ERG), immunohistochemistry, and quantitative real-time PCR analyses were performed. IOP and retinal thickness remained unchanged within all groups. The a-wave amplitudes were not altered in the ONA and ONA+C5-I groups, whereas a decrease was noted in ONA+C5-II animals (p < 0.05). ONA immunization provoked a significant decrease of the b-wave amplitude (p < 0.05), which could be preserved in ONA+C5-I, but not in ONA+C5-II animals. ONA animals showed a loss of RGCs (p = 0.001), while ONA+C5-I and ONA+C5-II retinae had similar cell counts as controls. A significant downregulation of apoptotic *Bax/Bcl2* mRNA was noted in ONA+C5-I retinae (p = 0.02). Significantly more C3^+^ and MAC^+^ cells were observed in ONA animals (p < 0.001). The amount of C3^+^ cells in both treatment groups was significantly increased (p < 0.01), while the number of MAC^+^ cells in the treated retinas did not differ from controls. The number of activated microglia cells remained unchanged in ONA animals, but was increased in the treatment groups (p < 0.05). Recoverin^+^ cells were diminished in ONA animals (p = 0.049), but not in treated ones. *Rho* mRNA was downregulated in ONA and in ONA+C5-II retinas (both p = 0.014). Less opsin^+^ cones were observed in ONA animals (p = 0.009), but not in the treated groups. Our results indicate that the C5 antibody inhibits activation of the complement system, preventing the loss of retinal function as well as RGC, cone bipolar, and photoreceptor loss. Therefore, this approach might be a suitable new treatment for glaucoma patients, in which immune dysregulation plays an important factor for the development and progression of glaucoma.

## Introduction

Glaucoma is a group of diseases of various causes with progressive optic nerve degeneration and loss of retinal ganglion cells (RGCs) with advancing visual field loss (Weinreb et al., 2014). It is the second most common cause of blindness worldwide (Quigley and Broman, 2006). Estimates assume that 76.9 million people worldwide will suffer from glaucoma in 2020, of which about 11 million will be blind in both eyes (Quigley and Broman, 2006). The severity of glaucoma damage often correlates with increased intraocular pressure (IOP), the major risk factor for the development and progression of this disease (Spaniol et al., 2015). However, one-sixth to one-third of patients with open-angle glaucoma show normal IOP levels. This form of glaucoma is called normal-pressure glaucoma (EGS, 2017). In these patients, other causes are suspected, such as impaired microcirculation (Shiga et al., 2016), elevated levels of glutamate (Harada et al., 2007) or nitric oxide (Polak et al., 2007), oxidative stress (Abu-Amero et al., 2006), circulatory disorders (Kashiwagi et al., 2001), or immunological changes (Bell et al., 2013). Disease-specific changes in complex autoantibody profiles were found in sera from glaucoma patients (Joachim et al., 2008; Reichelt et al., 2008). These altered immunoglobulin G (IgG) autoantibody patterns against ocular antigens were also identified in aqueous humor samples (Joachim et al., 2007a; Joachim et al., 2007b). Differences in the profiles of IgG autoantibodies between patients with normal-pressure glaucoma and patients with high pressure glaucoma were described (Tezel et al., 1998; Grus et al., 2004; Grus et al., 2006).

To identify immunological alterations IOP-independently, the experimental autoimmune glaucoma (EAG) model was developed (Wax et al., 2008). Here, an immunization with, for example, bovine optic nerve antigen (ONA) results in a complex systemic immune response without IOP elevation. The immune response comprises increasing serum antibody reactivity to multiple retinal and neuronal antigens that induce apoptosis of RGCs and optic nerve degeneration (Laspas et al., 2011; Joachim et al., 2013). Glial cells, particularly the microglia, play fundamental roles in local immune responses and immunosurveillance (Tezel and Fourth, 2009). These cells respond to neuronal stress or injury by adopting an activated state during glaucoma (Neufeld, 1999) and can also express complement factors (Luo et al., 2011; Rutar et al., 2011).

The complement system is a crucial component of the innate immune system. It can be activated *via* three distinct pathways, namely the classical, the lectin, and the alternative one. At the end, the membrane attack complex (MAC) is formed and generates a pore in the target cell resulting in cell lysis. In the last years, studies confirmed a contribution of the complement system in glaucoma disease. For example, depositions of complement components, like MAC, were observed in the human glaucomatous retina (Boehm et al., 2010; Tezel et al., 2010). Those depositions were also noted in ocular hypertension (OHT) animal models (Kuehn et al., 2006; Jha et al., 2011; Becker et al., 2015). In the EAG model, our group found an increase in the terminal complement components C3 and MAC in the retina and optic nerve of the animals 7 days after immunization with ONA (Reinehr et al., 2016a). This activation was even noted before a loss of RGCs and an optic nerve degeneration were observed.

Since the activation of the complement system seems to play a crucial role in glaucoma pathology, several studies in OHT models were performed in the last years altering the complement system. For example, a C1qa mutation protected DBA/2J mice from retinal and optic nerve degeneration (Howell et al., 2011; Howell et al., 2014). A lack of complement factor C5 in a mouse glaucoma model with elevated IOP reduced the severity of the glaucomatous damage in retina and optic nerve, suggesting that the inhibition of the complement factor C5 might be a future therapeutic approach also for patients (Howell et al., 2013).

The present study investigates whether the inhibition of the complement system can prevent the development and progression of glaucomatous damage in a glaucoma animal model without high IOP. To inhibit the complement system, we administered the monoclonal antibody BB5.1, which binds the complement factor C5, intravitreally. *In vivo* evaluations, such as spectral-domain optical coherence tomography (SD-OCT) and electroretinography (ERG) were performed in addition to immunohistology and quantitative real-time PCR (RT-qPCR). Our results indicate that the treatment led to a diminished complement activation, which resulted in preservation of RGCs and prevention of the loss of retinal function.

## Methods

### Animals

All procedures concerning animals adhered to the ARVO statement for the use of animals in ophthalmic and vision research. All experiments involving animals were approved by the animal care committee of North Rhine-Westphalia, Germany.

Male Lewis rats (Charles River, Sulzfeld, Germany), six weeks of age, were included in these experiments and kept under environmentally controlled conditions with free access to chow and water. Detailed observations and health checks with eye exams were performed regularly.

### Intravitreal Antibody Injection

The monoclonal antibody against the complement factor C5 BB5.1 (Hycult Biotech, Uden, Netherlands) was administered intravitreally in two different doses (15 µmol/µl: ONA+C5-I or 25 µmol/µl: ONA+C5-II) one day before immunization (**Supplementary Figure 1A**) (Copland et al., 2010). The intravitreal injections were repeated every 2 weeks. After being anesthetized with a ketamine/xylazine cocktail (100/4 mg/kg), eyes were dilated with tropicamide 5% and topically anesthetized with conjuncain. Under a stereomicroscope (Zeiss, Jena, Germany), 5 µl or 8 µl of BB5.1 were injected with a 32-gauge Hamilton cannula into the right eye, while the left eye served as untreated ONA group. After the injection, Floxal, an antibiotic ointment (Bausch&Lomb, Rochester, NY, USA), was applied to the eyes.

### Immunization

The preparation and immunization of ONA was carried out as previously described (Laspas et al., 2011; Joachim et al., 2013). Rats received an intraperitoneal injection with 8 mg/ml ONA mixed with incomplete Freund’s adjuvant (500 µl) plus 3 µg pertussis toxin (both Sigma Aldrich, St. Louis, MO, USA). The animals of the control group were injected with NaCl in Freund’s adjuvant and pertussis toxin. Booster injections with half of the original doses were given after 4 weeks. Animals were sacrificed 6 weeks after immunization (**Supplementary Figure 1A**).

### IOP Measurement

The IOP of the animals was measured one week before (baseline) and once every week after the immunization (**Supplementary Figure 1A**), always at the same time of day, with a rebound tonometer TonoLab (iCare, Oy, Finland) (n = 6–8 animals/group). The tonometer was held horizontally in front of the animal’s eye (Pease et al., 2006). Ten measurements per eye were recorded. From these values, the mean value was calculated (Laspas et al., 2011).

### Optical Coherence Tomography

SD-OCT (Spectralis OCT, Heidelberg, Germany) of the retinas was performed 6 weeks after immunization in all groups (n = 4–8 eyes/group). After being anesthetized with ketamine/xylazine, eyes were dilated with tropicamide and topically anesthetized with conjuncain. The animals were placed in front of the SD-OCT imaging device. We performed two cross-sectioned B scans on the periphery of the retina and one peripapillary. Peripapillary retinal thickness measurements were obtained by circular scans with scanning angle of 12°. Thickness of retinal layers were manually measured in an axis perpendicular to the individual layers using ImageJ (NIH; Bethesda, MD, USA) software. The middle of the retina as well as three equidistant measurements per side were performed. Therefore, seven measurements were used to calculate the mean value for each retina (Guo et al., 2010; Berger et al., 2014).

### Electroretinogram Measurements

Before performing the ERG recordings under dim red light, rats were dark adapted overnight. The function of the retina was monitored using full-field flash electroretinography (HMsERG system; OcuScience LLC, Rolla, MO, USA) 6 weeks after immunization (n = 6–8/group) (Schmid et al., 2014; Palmhof et al., 2018). Scotopic flash ERGs were recorded at 0.1, 0.3, 1, 3, 10, and 25 cd.s/m^2^. Signals obtained from the corneal surface were amplified, digitized, averaged, and stored using commercial software (ERGView 4.380R; OcuScience LLC) for later analysis. Data was filtered with 50 Hz before evaluating amplitudes and latencies of the a- and b-wave. Data was then transferred to a spreadsheet program (Excel; Microsoft Corp., Redmond, WA, USA) for statistical analysis.

### Tissue Preparation for Retinal Cross-Sections

After 6 weeks, eyes were fixed in 4% paraformaldehyde. Following sucrose treatment, they were embedded in Tissue Tek (Thermo Fisher Scientific, Schwerte, Germany). Cross-sections (10 µm thickness) were cut with a cryostat and mounted onto Superfrost Plus slides (both Thermo Fisher Scientific) for later histological and immunohistological analysis (**Supplementary Figure 1A**).

### Histological Staining and Morphometric Analysis

Cross-sections of the retinas were stained with hematoxylin and eosin (H&E) (6 sections/eye; n = 6–8 eyes/group). H&E staining was realized to investigate retinal thickness. After the H&E staining, all slides were dehydrated in ethanol following incubation in xylene before being mounted with Eukitt (O-Kindler GmbH & Co, Freiburg, Germany). Two pictures of each H&E stained retinal cross-sections, at a distance of 1500 µm dorsal and ventral to the optic nerve, were taken from each retina with a microscope equipped with a CCD camera (Axio Imager M1; Carl Zeiss Microscopy). The thickness of the ganglion cell complex (nerve fiber layer, ganglion cell layer (GCL), and inner plexiform layer) and solely the GCL was analyzed *via* a measuring tool in the Zen 2012 software (Zeiss). Briefly, the graphic menu tool was used to draw lines to measure the layers. The lengths were shown in µm. For each analysis, three measurements per picture were obtained and then averaged (Joachim et al., 2017).

### Immunohistology

In order to identify the different retinal cell types, specific antibodies were used for immunofluorescence staining (n = 6–8 eyes/group; **Table 1**; **Supplementary Figure 1B**). Briefly, retina sections were blocked with a solution containing 10-20% donkey and/or goat serum and 0.1% Triton-X in PBS. Primary antibodies were incubated at room temperature overnight. Incubation using corresponding secondary antibodies was performed for 1 h on the next day. Nuclear staining with 4′,6 diamidino-2-phenylindole (DAPI) was included to facilitate the orientation on the slides. Negative controls were performed by applying secondary antibodies only. All photographs were taken using an Axiocam HRc CCD camera on an Axio Imager M1 fluorescence microscope (Zeiss). Two photos of the peripheral and two of the central part of each retinal section were captured (Reinehr et al., 2018a). The images were then transferred to Corel Paint Shop Pro (Version 13, Corel Corporation, CA, USA) and equal excerpts were cut out. Brn-3a^+^, cleaved caspase 3^+^, C3^+^, MAC^+^, Iba1^+^, ED1^+^, recoverin^+^, PKCα^+^, and opsin^+^ cells were counted using ImageJ software. In regard to cleaved caspase 3^+^ and ED1^+^ cells, only cells co-localized with Brn-3a or Iba1, respectively, were taken into account. Group comparison was performed after transferring the data to Statistica software (Version 13; Dell, Tulsa, OK, USA).

**Table 1:** Primary and corresponding secondary antibodies applied for immunohistochemistry of retinal tissue.

Primary antibodies			Secondary antibodies		
Antibody	Company	Dilution	Antibody	Company	Dilution
Brn-3a	Santa Cruz	1:100	Donkey anti-goat Alexa Fluor 488	Dianova	1:500
C3	Cedarlane	1:500	Goat anti-rabbit IgG Cy 3	Linaris	1:500
C5b-9 (MAC)	Biozol	1:100	Goat anti-mouse Alexa Fluor 488	Life technology	1:500
Cleaved caspase 3	Sigma-Aldrich	1:300	Donkey anti-rabbit Alexa Fluor 555	Invitrogen	1:800
ED1	Millipore	1:250	Goat anti-mouse Alexa Fluor A488	Invitrogen	1:600
GFAP	Millipore	1:400	Donkey anti-chicken Cy3	Millipore	1:500
Iba1	Wako	1:500	Donkey anti-rabbit Alexa Fluor 555	Invitrogen	1:400
L-opsin	Millipore	1:200	Donkey anti-rabbit Alexa Fluor 555	Invitrogen	1:500
PKCα	Santa-Cruz	1:500	Goat anti-mouse Alexa Fluor 488	Life technology	1:500
Recoverin	Millipore	1:1000	Donkey anti-rabbit Alexa Fluor 555	Invitrogen	1:400
Rhodopsin	Abcam	1:400	Goat anti-mouse Alexa Fluor 488	Life technology	1:500
Vimentin	Sigma-Aldrich	1:500	Goat anti-mouse Alexa Fluor 488	Invitrogen	1:500

Measurement and analysis of rhodopsin, glial fibrillary acidic protein (GFAP), and vimentin labeled area were performed by using an ImageJ macro (Casola et al., 2016; Reinehr et al., 2018a). Briefly, images were transformed into grayscale. To minimize interference with background labeling, a rolling ball radius of 65 pixels for rhodopsin and 50 pixels for vimentin staining was subtracted. GFAP staining was processed without background subtraction. Then, for each picture, a suitable lower and upper threshold was determined. The ideal thresholds were obtained when the grayscale picture corresponded with the original one. Once all lower and upper thresholds from each picture were obtained, the mean value was calculated, and this number was then used for the final analysis. The percentage of the labeled area was then measured between these defined thresholds (rhodopsin: lower threshold: 7.86; upper threshold: 253.77; GFAP: lower threshold: 9.53; upper threshold: 265; vimentin: lower threshold: 3.15; upper threshold: 263).

### Retinal Quantitative Real-Time PCR

Retinas of each animal were used for RNA preparation and cDNA synthesis as previously described (n = 5 eyes/group) (Reinehr et al., 2016a; Reinehr et al., 2018b). The designed oligonucleotides for RT-qPCR are shown in **Table 2**. The RT-qPCR was performed using DyNAmo Flash SYBR Green (Thermo Scientific) on the PikoReal RT-qPCR Cycler (Thermo Scientific). The primer concentration was optimized to a final concentration of 200 nM and combined with 200 ng of retinal RNAs per well. We prepared two reactions per RNA sample (duplicates) with a final volume of 20 µl per reaction. The average threshold cycle values of the two independent experiments were used to calculate the ratios for the target genes. The normal threshold cycle values for the reference genes (*β-actin* and *Cyclophilin*) were used for normalization.

**Table 2:** Sequences of oligonucleotide pairs.

Gene	Forward (F) and reverse (R) oligonucleotides	GenBank acc. no.	Amplicon size
*β-actin*-F	cccgcgagtacaaccttct	NM_031144.3	72 bp
*β-actin*-R	cgtcatccatggcgaact		
*Bax*-F	gctggacactggacttcctc	NM_017059.2	127 bp
*Bax*-R	actccagccacaaagatggt		
*Bcl-2*-F	gtacctgaaccggcatctg	NM_016993.1	76 bp
*Bcl-2*-R	ggggccatatagttccacaa		
*C3*-F	tcgaaatccctcccaagtc	NM_016994.2	60 bp
*C3*-R	cgatcttcaaggggacaatg		
*C5*-F	tctcaggccaaagagagacc	XM001079130.4	73 bp
*C5*-R	acggtgtttgtatttagcagctt		
*Cd68*-F	ctcacaaaaaggctgccact	NM_001031638.1	99 bp
*Cd68*-R	ttccggtggttgtaggtgtc		
*Cyclophilin*-F	tgctggaccaaacacaaatg	M19553.1	88 bp
*Cyclophilin*-R	cttcccaaagaccacatgct		
*Gfap*-F	tttctccaacctccagatcc	NM_017009.2	64 bp
*Gfap*-R	gaggtggccttctgacacag		
*Iba1*-F	ctccgaggagacgttcagtt	XM_006256063.3	96 bp
*Iba1*-R	tttttctcctcatacatcagaatcatcagaat		
*Il1b*-F	tgtgatgaaagacggcacac	NM_031512.2	70 bp
*Il1b*-R	cttcttctttgggtattgtttgg		
*Pou4f1*-F	ctggccaacctcaagatcc	XM_008770931.2	72 bp
*Pou4f1*-R	cgtgagcgactcgaacct		
*Rho*-F	accttgagggcttctttgc	NM_033441.1	70 bp
*Rho*-R	tcaatggccaggactacca		

F, forward; R, reverse; bp, base pair. The listed primer pairs were used in quantitative real-time PCR experiments, while β-actin and Cyclophilin served as housekeeping genes. The predicted amplicon sizes are given.

### Statistics

Data are presented as mean ± standard deviation (SD), unless otherwise noted. In regard to IOP and ERG recordings, OCT analysis, histology measurements, and immunohistology analysis, the groups were compared *via* one-factorial ANOVA followed by Tukey post-hoc test using Statistica software. For H&E, control values were set to 100%. Regarding RT-qPCR, the relative expression values are presented as median ± quartile+minimum/maximum and were assessed *via* Pair Wise Fixed Reallocation Randomisation Test using REST^©^ software (Qiagen, Hilden, Germany) (Pfaffl et al., 2002). p-Values below 0.05 were considered statistically significant, with ONA vs. control: *p < 0.05, **p < 0.01, ***p < 0.001 and ONA+C5-II vs. control: #p < 0.05, ##p < 0.01, ###p < 0.001.

## Results

### No Alterations in Intraocular Pressure

**Figure 1:**
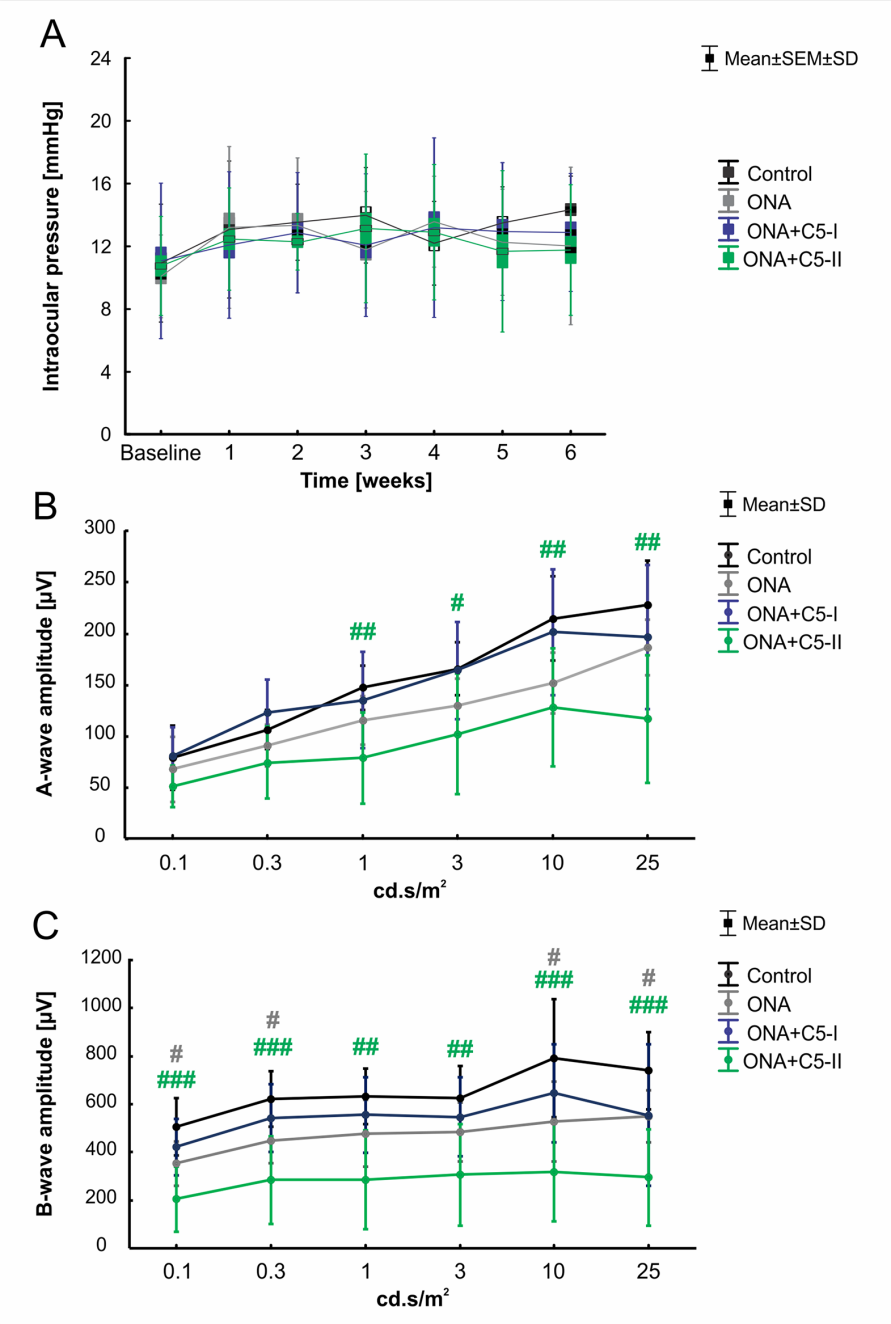
Unaltered intraocular pressure and improved retinal function after C5 administration. **(A)** The IOP of the animals was measured before (baseline) and once every week after the immunization. No significant differences between the control, the ONA, and both treatment groups were observed at all points in time. **(B)** The a-wave amplitudes of the ERG measurements at all light intensities. At 1-25 cd.s/m^2^, a significant decrease was noted in ONA+C5-II animals compared to controls (p < 0.05). **(C)** The b-wave amplitudes of the ERG measurements at all light intensities. A significant lower amplitude was observed in ONA retinae compared to controls at 0.1, 0.3, 10, and 25 cd.s/m^2^ (p < 0.05). At all light intensities, the b-wave amplitude of ONA+C5-II was significantly decreased in comparison to controls (p < 0.01). Values for IOP are mean ± SEM ± SD. Values for ERG are mean ± SD. Grey^# ^= ONA vs control; green^# ^= ONA+C5-II vs control. ^#^p < 0.05, ^##^p < 0.01, ^###^p < 0.001.

Statistical evaluation of the IOP (n = 6–8/group) at the beginning of the study (baseline) showed no significant differences between the control group, the ONA group (p = 0.81), and both treatment groups (both: p = 0.99). IOPs remained stable throughout the study. Also, six weeks after immunization, no statistically significant differences were found in all groups compared to controls (ONA: p = 0.11; ONA+C5-I: p = 0.45; ONA+C5-II: p = 0.09; **Table 3**; **Figure 1A**).

**Table 3:** Values of intraocular pressure (IOP) throughout the study.

	Mean IOP						
	Baseline	Week 1	Week 2	Week 3	Week 4	Week 5	Week 6
**Control**	10.9 ± 1.8	13.1 ± 2.2	13.5 ± 1.2	13.9 ± 1.5	12.2 ± 1.3	13.5 ± 1.1	14.3 ± 1.1
**ONA**	10.1 ± 1.3	13.2 ± 2.6	13.3 ± 2.2	11.8 ± 1.9	13.6 ± 1.4	12.3 ± 1.7	12.0 ± 2.5
**P-value**	0.81	0.99	0.99	0.15	0.54	0.58	0.11
**ONA+C5-I**	11.1 ± 2.5	12.1 ± 2.3	12.8 ± 1.9	12.1 ± 2.3	13.2 ± 2.9	12.9 ± 2.2	12.9 ± 1.9
**P-value**	0.99	0.82	0.85	0.25	0.76	0.94	0.45
**ONA+C5-II**	10.8 ± 1.6	12.5 ± 1.6	12.3 ± 0.9	13.1 ± 2.4	12.9 ± 2.2	11.7 ± 2.6	11.8 ± 2.1
**P-value**	0.99	0.96	0.53	0.87	0.92	0.32	0.09

Values are mean ± SD.

### Improved B-Wave Amplitude After Intravitreal C5 Antibody Administration

ERG measurements were performed 6 weeks after immunization (n = 6–8/group; **Table 4**; **Figures 1B, C**). Regarding the a-wave amplitudes, which reflect the electrical output of the photoreceptors, no alterations were noted within all groups at 0.1 and 0.3 cd.s/m^2^ (p > 0.05). At 1-25 cd.s/m^2^, no changes were noted in ONA (p > 0.05) and ONA+C5-I (p > 0.05) animals compared to controls, while a significant decrease of the a-wave amplitude was observed in ONA+C5-II retinas (p < 0.05).

**Table 4:** Summary of ERG results.

	A-wave amplitude (µV)					
	0.1 cd.s/m^2^	0.3 cd.s/m^2^	1 cd.s/m^2^	3 cd.s/m^2^	10 cd.s/m^2^	25 cd.s/m^2^
**Control**	79.9 ± 11.0	106.1 ± 6.7	147.0 ± 7.6	165.3 ± 9.0	214.4 ± 14.6	227.6 ± 15.1
**ONA**	69.2 ± 11.1	88.1 ± 7.9	107.1 ± 8.9	120.2 ± 9.9	148.4 ± 10.5	176.9 ± 9.6
**P-value**	0.90	0.58	0.15	0.18	0.06	0.22
**ONA-C5-I**	84.7 ± 11.7	122.8 ± 14.1	133.5 ± 21.0	165.1 ± 21.2	197.7 ± 27.1	196.3 ± 28.5
**P-value**	0.98	0.74	0.92	0.99	0.94	0.72
**ONA-C5-II**	51.1 ± 7.0	74.1 ± 34.8	78.5 ± 15.6	102.0 ± 20.9	128.1 ± 20.3	116.5 ± 21.9
**P-value**	0.23	0.13	**0.005**	**0.03**	**0.009**	**0.001**
	**B-wave amplitude (µV)**					
**0.1 cd.s/m^2^**	**0.3 cd.s/m** **^2^**	**1 cd.s/m** **^2^**	**3 cd.s/m** **^2^**	**10 cd.s/m** **^2^**	**25 cd.s/m^2^**
**Control**	506.9 ± 42.5	622.5 ± 40.9	632.5 ± 41.3	625.1 ± 47.2	792.2 ± 86.9	740.0 ± 56.8
**ONA**	323.3 ± 32.8	410.9 ± 34.8	415.5 ± 46.7	428.6 ± 41.5	446.5 ± 50.8	496.3 ± 34.2
**P-value**	**0.02**	**0.03**	0.05	0.10	**0.01**	**0.04**
**ONA-C5-I**	425.9 ± 52.2	542.7 ± 62.8	553.6 ± 71.0	545.0 ± 73.3	648.5 ± 91.6	640.6 ± 84.9
**P-value**	0.65	0.76	0.83	0.83	0.63	0.74
**ONA-C5-II**	204.8 ± 47.8	283.8 ± 64.6	285.9 ± 72.9	306.6 ± 74.8	316.1 ± 72.2	294.6 ± 71.2
**P-value**	**0.0003**	**0.0004**	**0.001**	**0.003**	**0.0007**	**0.0002**

For all light intensities, the mean a- and b-wave amplitudes of all groups and the respective p-values are shown. Significant p-values are marked in bold. Values are mean ± SD.

The b-wave amplitude represents the output of the inner nuclear layers of the retina. At the light intensities of 0.1 and 0.3 cd.s/m^2^, a significant reduction was detected in ONA animals (p < 0.05) and in the ONA+C5-II group (p < 0.001). However, no changes were noted in the ONA+C5-I treated retinas (p > 0.05). At 1 and 3 cd.s/m^2^, only a significant decrease of the b-wave amplitude of the ONA+C5-II animals was seen (p < 0.01), while it was not altered in ONA and ONA+C5-I retinas (p > 0.05). A significant reduction of the b-wave amplitude was observed in ONA retinas (p < 0.05), but not in the ONA+C5-I ones (p > 0.05) at 10 and 25cd.s/m^2^. Again, significantly decreased amplitudes were measured in ONA+C5-II animals (p < 0.001).

### Comparable Retinal Thickness in All Groups

**Figure 2:**
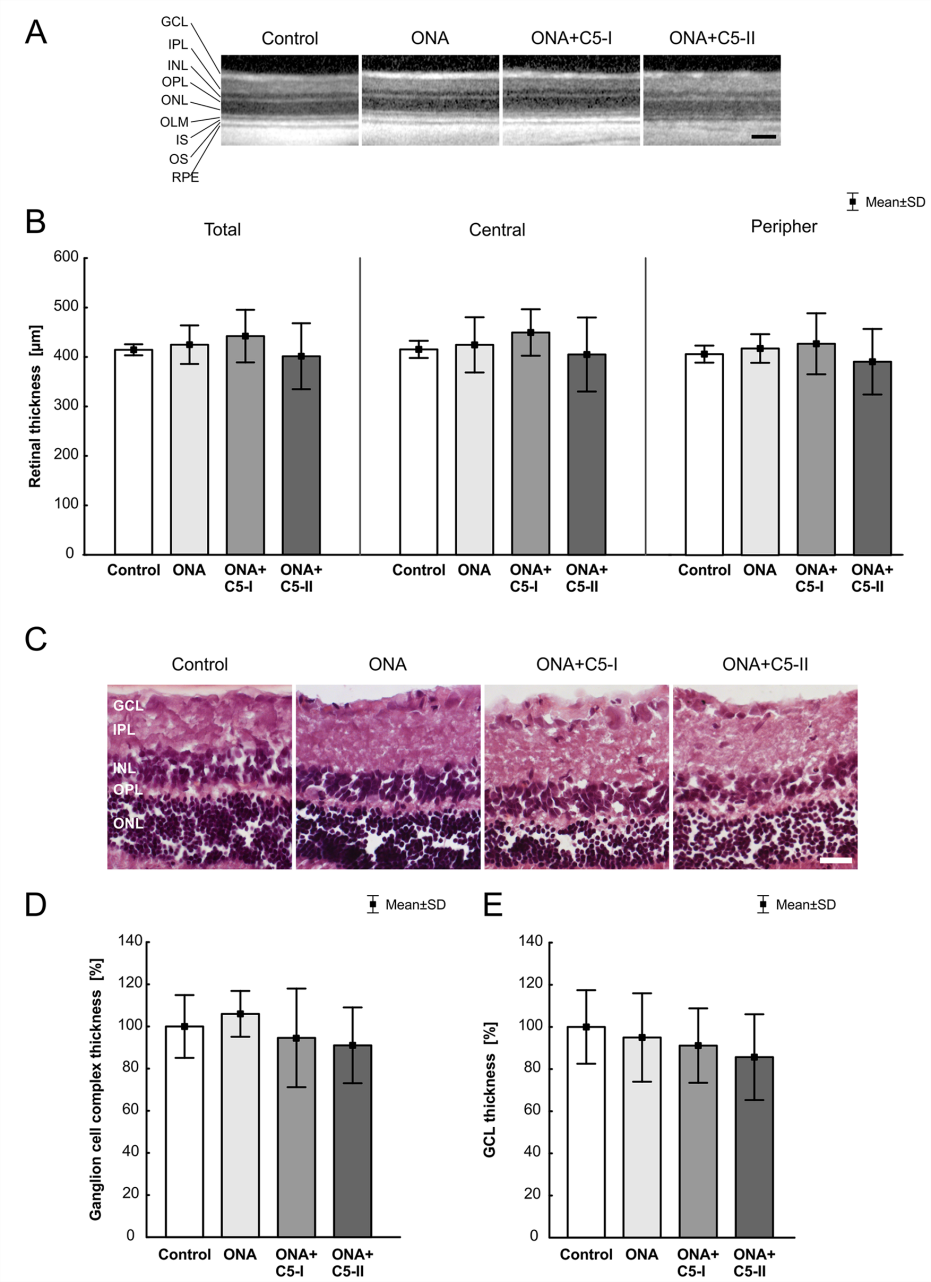
Unchanged retinal thickness. **(A)** After 6 weeks, SD-OCT pictures of all groups showed no visual abnormalities. **(B)** There were no significant differences in the thickness of the total, central and peripheral retina between all groups. **(C)** H&E stained retinal cross-sections of all groups displayed an intact retinal morphology. **(D)** No alterations in the thickness of the ganglion cell complex between all groups were observable. **(E)** Also, the average thickness of the GCL was similar in all four groups. GCL, ganglion cell layer; IPL, inner plexiform layer; INL, inner nuclear layer; OPL, outer plexiform layer; ONL, outer nuclear layer; IS, inner segment; OS, outer segment; RPE, retinal pigment epithelium. Values are mean ± SD. Scale bars = (A) 200 µm, (C) 50 µm.

6 weeks after immunization, there were no changes in the total retina thickness, when evaluated *via* SD-OCT (n = 4–8/group), in ONA retinae compared to controls (p = 0.97). The measurements of both treatment groups also revealed a comparable total thickness compared to the control animals (ONA+C5-I: p = 0.83; ONA+C5-II; p = 0.94; **Table 5**; **Figures 2A, B**). Furthermore, the central retina thickness showed no differences between all groups (ONA: p = 0.99, ONA+C5-I: p = 0.81, ONA+C5-II: p = 0.98). Also, the peripheral thickness of the retina was not altered in ONA (p = 0.96) as well as in ONA+C5-I (p = 0.92) and ONA+C5-II animals (p = 0.92; **Figures 2A, C**). However, two eyes of the ONA+C5-II group could not be analyzed due to a lens opacification.

**Table 5:** Retinal thickness measured with optical coherence tomography (SD-OCT) and histological staining (H&E).

Total retinal thickness	SD-OCT [µm]	Central retinal thickness	SD-OCT [µm]	Peripheral retinal thickness	SD-OCT [µm]	Ganglion cell complex	H&E [%]	Ganglion cell layer	H&E [%]
**Control**	410.9 ± 11.1	**Control**	415.4 ± 17.3	**Control**	406.5 ± 17.2	**Control**	100.0 ± 14.9	**Control**	100.0 ± 17.5
**ONA**	421.3 ± 38.8	**ONA**	424.6 ± 55.9	**ONA**	417.9 ± 28.9	**ONA**	105.9 ± 10.9	**ONA**	94.9 ± 20.9
**P-value**	0.97	**P-value**	0.98	**P-value**	0.96	**P-value**	0.90	**P-value**	0.95
**ONA+C5-I**	438.5 ± 55.8	**ONA+C5-I**	449.6 ± 46.9	**ONA-C5-I**	427.5 ± 61.6	**ONA-C5-I**	94.6 ± 23.4	**ONA+C5-I**	91.2 ± 17.7
**P-value**	0.83	**P-value**	0.81	**P-value**	0.92	**P-value**	0.92	**P-value**	0.79
**ONA+C5-II**	398.1 ± 66.1	**ONA+C5-II**	405.1 ± 78.9	**ONA-C5-II**	391.2 ± 23.4	**ONA-C5-II**	91.0 ± 17.9	**ONA+C5-II**	85.7 ± 20.3
**P-value**	0.94	**P-value**	0.98	**P-value**	0.92	**P-value**	0.73	**P-value**	0.45

Values are mean ± SD.

The measurements of the H&E-stained ganglion cell complex in retinal cross-sections showed similar results. Here, the ONA (p = 0.90), the ONA+C5-I (p = 0.92), the ONA+C5-II (p = 0.73), and the control group displayed comparable values (**Table 5**; **Figures 2C, D**). Also, the average thickness of the GCL was quite similar in all groups (ONA: p = 0.95, ONA+C5-I: p = 0.79, ONA+C5-II: p = 0.45; **Table 5**; **Figures 2C, E**).

### Treatment Led to a Diminished Complement Activation

**Figure 3:**
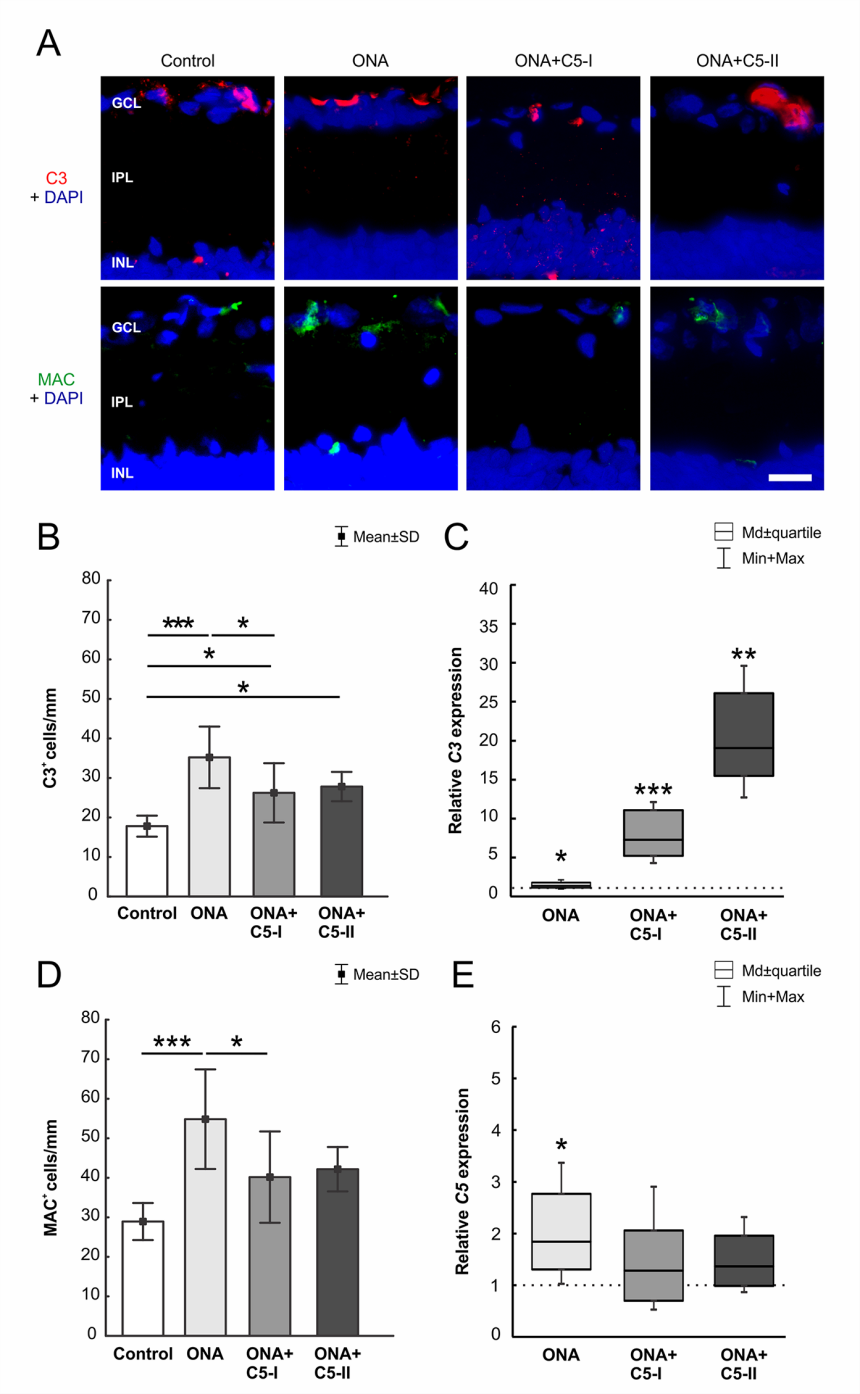
C5 antibody injection led to a diminished terminal pathway. **(A)** The complement factors C3 (red) and MAC (green) labeled in retinas at 6 weeks. Cell nuclei were stained with DAPI (blue). **(B)** Significantly more C3^+^ cells could be observed in ONA animals compared to controls (p = 0.0002). The number of C3^+^ cells in the treated groups was also significantly increased compared to controls (ONA+C5-I: p = 0.04, ONA+C5-II: p = 0.03). **(C)** Via RT-qPCR analyses, a significant upregulation of *C3* mRNA was detected in ONA retinas (p = 0.03) as well as in ONA+C5-I (p < 0.001) and ONA+C5-II animals (p = 0.002). **(D)** More MAC^+^ cells were detected in ONA animals compared to controls (p < 0.001). The number of MAC^+^ cells in the treated groups (ONA+C5-I and II) was not significant different to the controls. The counts in the ONA+C5-I group were significantly decreased compared to the ONA group (p = 0.03). **(E)** RT-qPCR analyses revealed an upregulation of *C5* mRNA in ONA animals compared to controls (p = 0.03). However, no changes were noted in both treated groups. Values for immunostaining are mean ± SD. Values for RT-qPCR are median ± quartile ± maximum/minimum. The dotted line in C and E represents the relative expression level of the control group. GCL, ganglion cell layer; IPL, inner plexiform layer; INL, inner nuclear layer. Scale bar = 20 μm. *p < 0.05, **p < 0.01, ***p < 0.001.

Significantly more C3^+^ cells (n = 6–8/group) were observed in ONA retinas compared to controls (p = 0.0002; **Table 6**; **Figures 3A, B**). Also, the number of C3^+^ cells was significantly increased in the treated groups compared to the control group (ONA+C5-I: p = 0.04; ONA+C5-II: p = 0.03). Additionally, a significant upregulation of *C3* mRNA (n = 5/group) was detected in ONA retinas compared to controls (1.4-fold, p = 0.03). Furthermore, a significant increase of *C3* mRNA expression levels was noted in ONA+C5-I (7.3-fold expression, p < 0.001) and ONA+C5-II retinas (19.07-fold expression, p = 0.002; **Figure 3C**).

**Table 6:** Histological analyses in the retina.

	Brn-3a^+^ cells/mm	Cleaved caspase^+^/Brn-3a^+^ cells [%]	C3^+^ cells/mm	MAC^+^ cells/mm	Recoverin^+^ cells/mm	PKCα^+^ cells/mm	Rhodopsin^+^ area [%]/image	L-opsin^+^ cells/mm	Iba1^+^ cells/mm	ED1^+^ and Iba1^+^ cells/mm	GFAP^+^ area [%]/image	Vimentin^+^ area [%]/image
**Control**	49.7 ± 5.5	16.6 ± 10.2	17.8 ± 2.7	28.9 ± 4.7	33.8 ± 7.7	103.8 ± 13.2	9.5 ± 2.1	66.2 ± 5.6	8.1 ± 1.6	1.40 ± 1.0	6.9 ± 3.2	4.8 ± 2.1
**ONA**	20.8 ± 12.9	11.6 ± 11.6	35.2 ± 7.8	54.8 ± 12.6	22.7 ± 10.3	86.7 ± 19.7	7.2 ± 2.7	56.4 ± 6.7	14.1 ± 6.4	6.8 ± 5.1	9.9 ± 2.9	7.8 ± 4.2
**P-value**	**0.001**	0.87	**0.0002**	**0.0003**	**0.049**	0.20	0.16	**0.01**	0.28	**0.22**	0.28	0.30
**ONA-C5-I**	36.3 ± 15.3	17.0 ± 14.2	26.2 ± 7.5	40.2 ± 11.6	29.7 ± 2.5	102.9 ± 13.9	9.8 ± 1.3	62.9 ± 5.8	21.9 ± 8.7	11.8 ± 7.8	9.9 ± 1.9	5.9 ± 3.6
**P-value**	0.22	0.99	**0.04**	0.10	0.69	0.99	0.99	0.66	**0.001**	**0.004**	0.22	0.90
**ONA-C5-II**	39.4 ± 17.9	21.2 ± 16.2	27.8 ± 3.7	42.2 ± 5.6	34.9 ± 8.4	104.2 ± 15.5	7.8 ± 2.1	61.0 ± 4.1	16.6 ± 5.2	9.0 ± 4.1	7.5 ± 3.9	5.9 ± 1.9
**P-value**	0.50	0.91	**0.03**	0.09	0.99	0.99	0.40	0.34	0.07	**0.047**	0.98	0.92

Values are mean ± SD. Significant p-values are marked in bold.

Regarding MAC staining (n = 6–8/group), more MAC^+^ cells were present in ONA animals compared to control retinas (p = 0.0003; **Table 6**; **Figures 3A, D**). However, the amount of MAC^+^ cells in the treated groups was comparable to controls (ONA+C5-I: p = 0.10; ONA+C5-II: p = 0.09). A significant lower cell number was observed in ONA+C5-I animals in comparison to the ONA group (p = 0.03). In addition, RT-qPCR analyses (n = 5/group) revealed an upregulation of *C5* mRNA in ONA animals in contrast to controls (1.84-fold expression, p = 0.03; **Figure 3E**). However, no changes were noted in ONA+C5-I (1.29-fold expression, p = 0.4) as well as in ONA+C5-II retinas (1.36-fold expression, p = 0.1), when compared to controls.

### Protection of RGCs After Intravitreal C5 Antibody Injection

**Figure 4:**
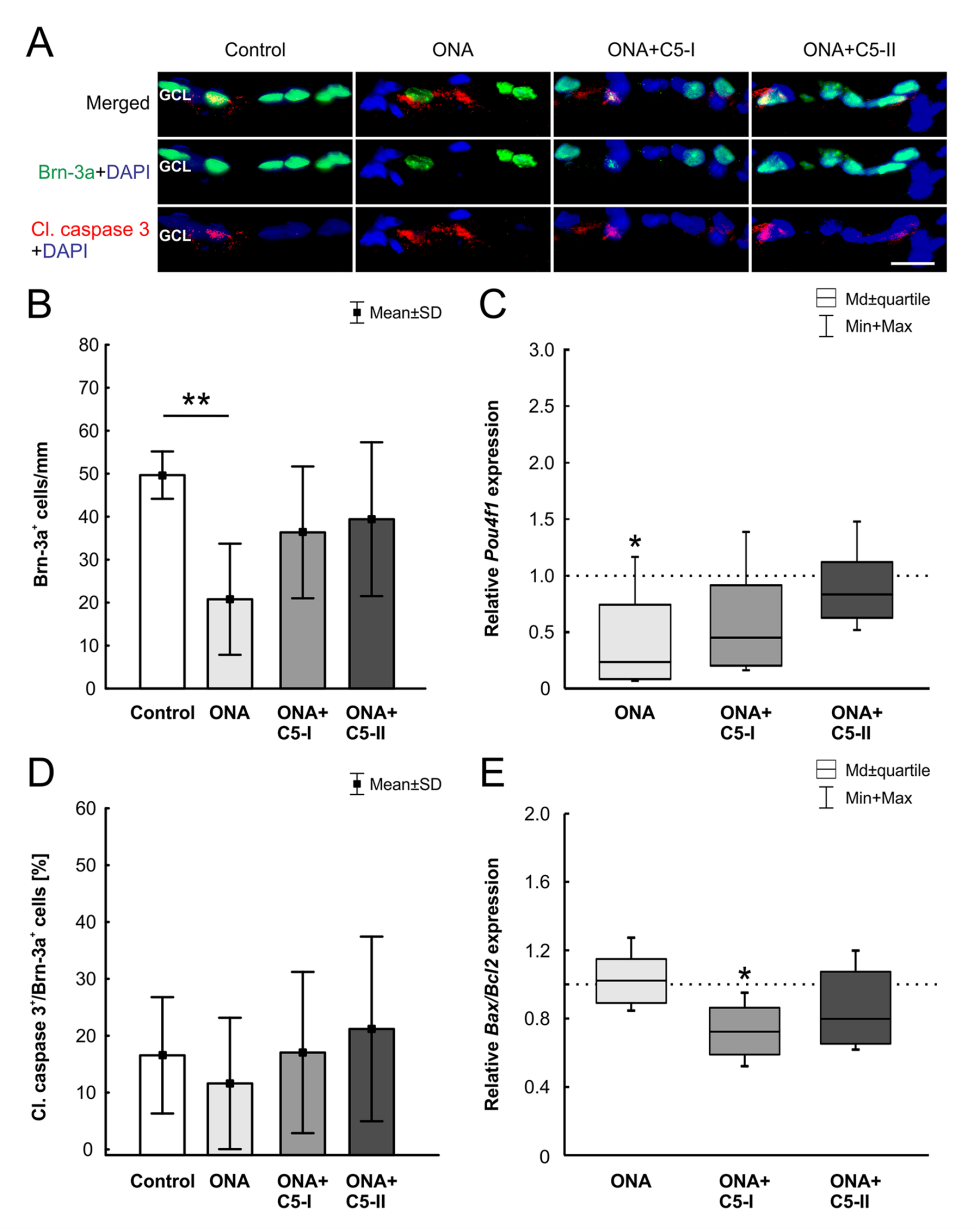
Protection of RGCs after intravitreal treatment. **(A)** The RGCs were labeled with Brn-3a (green) and activated caspase-3 (red) was used to stain the cytoplasm of apoptotic cells. DAPI (blue) counterstained cell nuclei. **(B)** In the ONA group the number of Brn-3a^+^ cells decreased significantly (p = 0.001). The analysis of the treated groups, ONA+C5-I and II, showed no changes compared to the control group (p > 0.05). **(C)** In RT-qPCR analyses, lower *Pou4f1* mRNA levels were found in ONA immunized eyes compared to controls (p = 0.03). There was no significant difference between the control and both treatment groups. **(D)** There were also no alterations in the proportion of the RGC undergoing apoptosis between all groups. **(E)** In RT-qPCR analyses, we noted no difference between control and ONA animals regarding *Bax/Bcl2* mRNA expression levels. However, a significant downregulation of *Bax/Bcl2* mRNA was observed in ONA+C5-I retinas (p = 0.02). No changes were noted in the ONA+C5-II group. Values for immunostaining are mean ± SD. Values for RT-qPCR are median ± quartile ± maximum/minimum. The dotted line in C and E represents the relative expression level of the control group. GCL, ganglion cell layer. Scale bar = 20 μm. *p < 0.05, **p < 0.01.

After 6 weeks, a significantly reduced number of Brn-3a^+^ RGCs (n = 6–8/group) was noted in the ONA group in comparison to controls (p = 0.001; **Table 6**; **Figures 4A, B**). In the treated groups, the RGC count was not significantly different compared to the control group (ONA+C5-I: p = 0.22; ONA+C5-II: p = 0.5). In RT-qPCR analyses (n = 5/group), we found lower *Pou4f1* mRNA levels in ONA eyes compared to controls (0.24-fold expression, p = 0.03; **Figure 4C**). In accordance with immunohistological results, there was no difference between controls and both treatment groups in *Pou4f1* mRNA expression (ONA C5-I: 0.45-fold expression, p = 0.1; ONA C5-II: 0.84-fold expression, p = 0.25).

No alterations in the proportion of the RGC undergoing apoptosis between all groups (n = 6–8/group) were noted (ONA p = 0.87, ONA+C5-I p = 1.00, ONA+C5-II p = 0.91; **Table 6**; **Figure 4A, D**). In RT-qPCR analyses (n = 5/group), we observed no changes in *Bax/Bcl2* mRNA expression levels in ONA animals (1.02-fold expression, p = 0.8; **Figure 4E**), when compared to controls. However, a significant downregulation of *Bax/Bcl2* mRNA was observed in ONA+C5-I retinas compared to controls (0.72-fold expression, p = 0.02). The *Bax/Bcl2* mRNA expression levels in the ONA+C5-II group was not significantly different from the control group (0.8-fold expression, p = 0.14).

### Complement Inhibition Had Little Effect on Glia Cells and Cytokine Response

**Figure 5:**
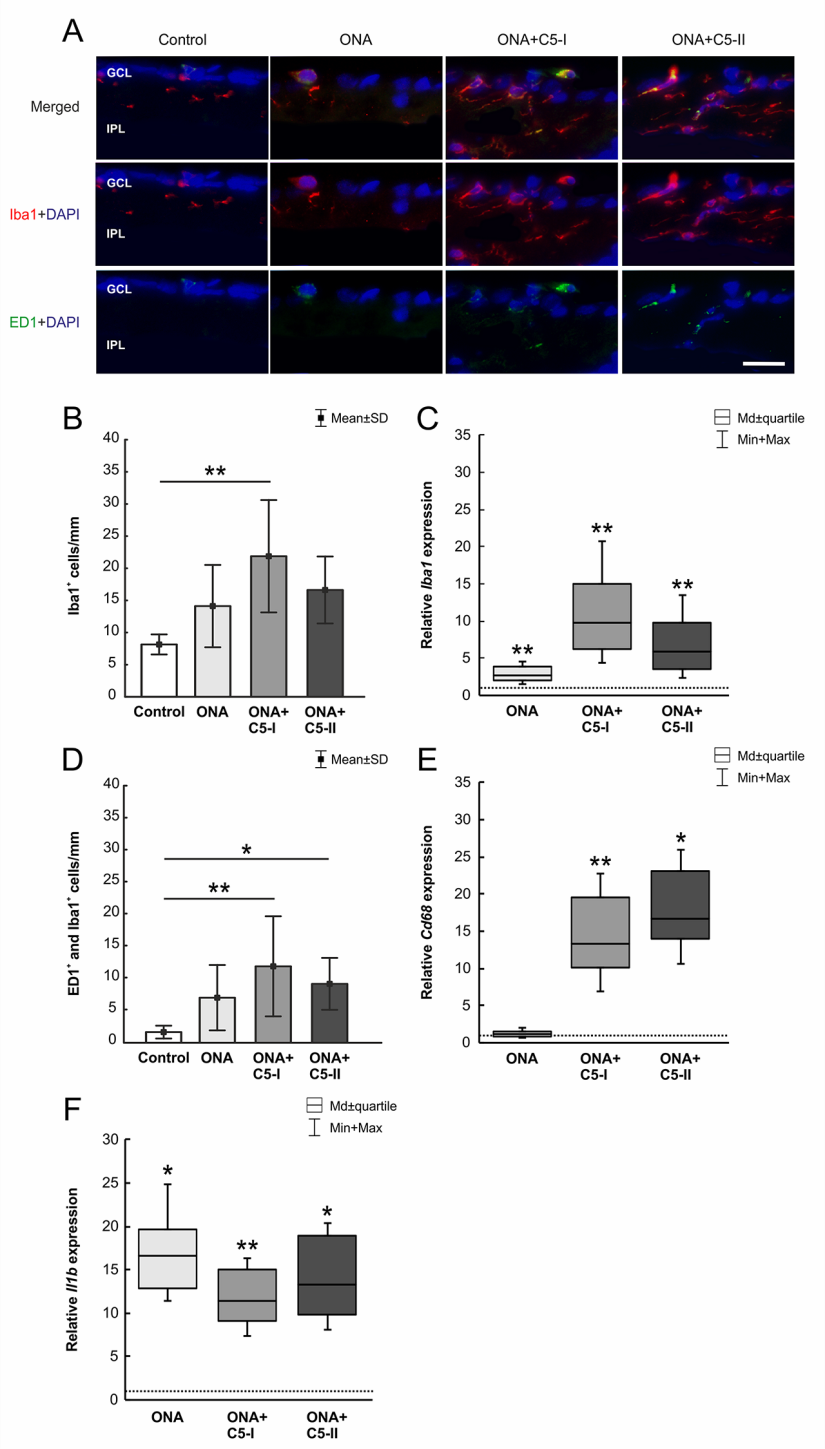
Little effects on microglia cells. **(A)** The total number of microglia was labeled with Iba1 (red) and activated microglia were additionally marked with ED1 (green). DAPI counterstained cell nuclei (blue). **(B)** Iba1^+^ microglia numbers were not altered in ONA and ONA+C5-II animals. However, a significant increase of Iba1^+^ cells was noted in ONA+C5-II retinae (p = 0.001). **(C)** The mRNA expression levels of *Iba1* showed a significant upregulation in ONA retinas (p = 0.009) as well as in both treatment groups (ONA+C5-I: p = 0.002; ONA+C5-II: p = 0.009). **(D)** The number of activated (ED1^+^ and Iba1^+^) microglia remained unchanged in the ONA group. A significant increase in numbers was observable in ONA+C5-I (p = 0.004) as well as in ONA+C5-II animals (p = 0.047) in comparison to the controls. **(E)** *Cd68* mRNA levels remained unchanged in ONA animals compared to controls. However, a significant upregulation was revealed in ONA+C5-I (p = 0.004) and in ONA+C5-II retinas (p = 0.03). **(F)** The *Il1b* mRNA expression was upregulated in ONA retinas compared to controls (p = 0.01) as well as in ONA+C5-I (p = 0.009) and ONA+C5-II animals (p = 0.02). Values for immunostaining are mean ± SD. Values for RT-qPCR are median ± quartile ± maximum/minimum. The dotted line in C, E, and F represents the relative expression level of the control group. GCL, ganglion cell layer; IPL, inner plexiform layer. Scale bar = 20 μm. *p < 0.05, **p < 0.01.

The number of Iba1^+^ microglia (n = 6–8/group) was not altered in ONA animals compared to controls (p = 0.28; **Table 6**; **Figures 5A, B**). However, a significant increase in the number of Iba1^+^ cells was observed in the ONA+C5-I group (p = 0.001). No changes were noted in ONA+C5-II retinas compared to controls (p = 0.07). The mRNA expression levels of *Iba1* (n = 5/group) showed a significant upregulation in ONA retinas (2.68-fold expression; p = 0.009; **Figure 5C**). Also, in both treatment groups, a significant upregulation of *Iba1* expression levels were noted (ONA+C5-I: 9.84-fold expression; p = 0.002; ONA+C5-II: 5.84-fold expression; p = 0.009).

In regard to ED1^+^ and Iba1^+^ activated microglia (n = 6–8/group), no alterations were detected in the ONA group compared to controls (p = 0.22; **Table 6**; **Figures 5A, D**). Significantly more active microglia were observed in ONA+C5-I (p = 0.004) as well as in ONA+C5-II animals (p = 0.047) in comparison to the controls. The mRNA levels of *Cd68* (n = 5/group) remained unchanged in ONA animals compared to controls (1.20-fold expression; p = 0.3; **Figure 5E**). A significant upregulation of *Cd68* mRNA expression was revealed in ONA+C5-I (13.33-fold expression; p = 0.004) and in ONA+C5-II retinas (16.74-fold expression; p = 0.03).

The *Il1b* mRNA expression (n = 5/group) was upregulated in ONA retinas compared to controls (16.56-fold expression; p = 0.01; **Figure 5F**). Furthermore, analyses of *Il1b* expression revealed a significant upregulation in ONA+C5-I (11.44-fold expression; p = 0.009) and ONA+C5-II animals (13.33-fold expression; p = 0.02).

**Figure 6:**
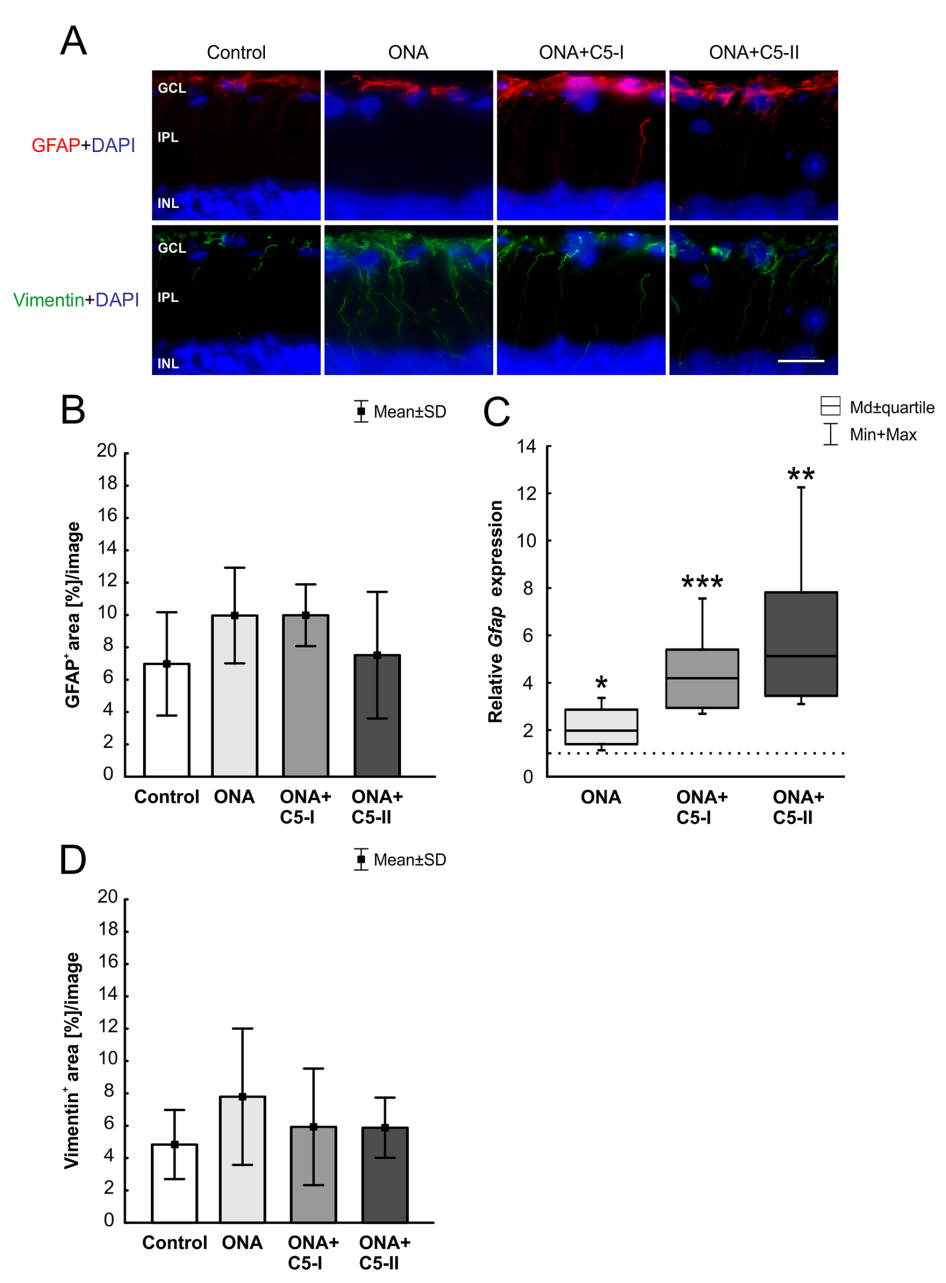
Unaltered macroglia response. **(A)** Astrocytes were labeled with GFAP (red) and Müller glia with vimentin (green). Cell nuclei were stained with DAPI (blue). **(B)** The GFAP^+^ area was similar in all groups. **(C)** However, a significant upregulation of *Gfap* mRNA levels was revealed in ONA (p = 0.01) as well as in ONA+C5-I (p < 0.001) and in ONA+C5-II retinae (p = 0.001). **(D)** No changes in vimentin^+^ staining area were observed among all groups. Values for immunostaining are mean ± SD. Values for RT-qPCR are median ± quartile ± maximum/minimum. The dotted line in C represents the relative expression level of the control group. GCL, ganglion cell layer; IPL, inner plexiform layer; INL, inner nuclear layer. Scale bar = 20 µm. *p < 0.05, **p < 0.01, ***p < 0.001.

The GFAP^+^ area (n = 6–8/group) was similar between ONA and control animals (p = 0.28; **Table 6**; **Figures 6A, B**). Also, no changes were detectable in ONA+C5-I (p = 0.22) and ONA+C5-II animals (p = 0.99) compared to controls. Interestingly, the mRNA expression levels of *Gfap* (n = 5/group) were significantly increased in ONA retinas (1.98-fold expression; p = 0.01; **Figure 6C**) as well as in both treatment groups (ONA+C5-I: 4.19-fold expression, p < 0.001; ONA+C5-II: 5.13-fold expression, p = 0.001).

The evaluation of the vimentin^+^ area (n = 6–8/group) revealed no alterations among all groups (**Table 6**; **Figures 6A, D**). No changes were noted between the stained area of the control, the ONA (p = 0.30), the ONA+C5-I (p = 0.90), and the ONA+C5-II animals (p = 0.92).

### Preservation of Cone Bipolar Cells Through Treatment

**Figure 7:**
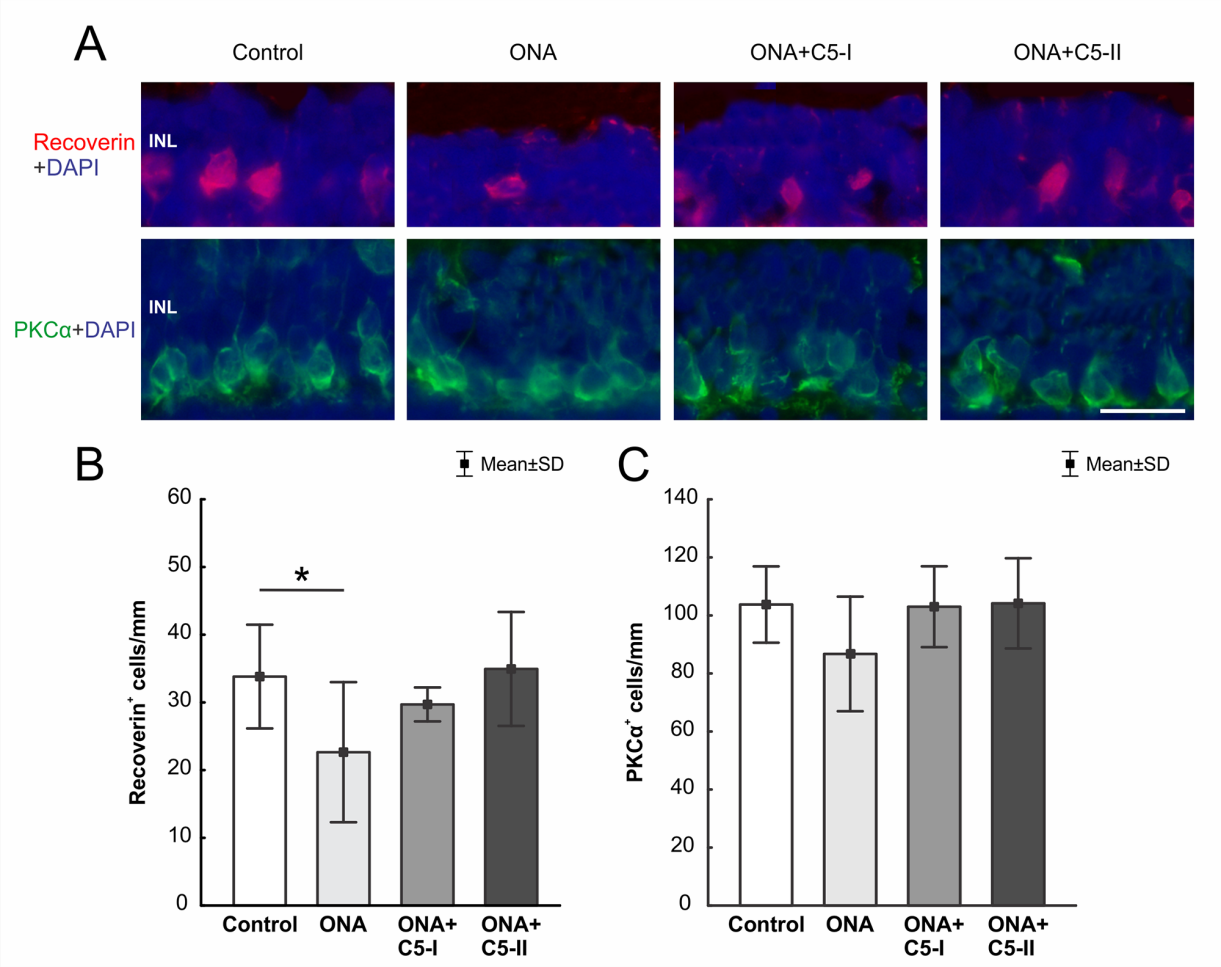
Preservation of cone bipolar cells. **(A)** Cone bipolar cells were labeled with recoverin (red) and rod bipolar cells with PKCα (green). DAPI stained cell nuclei (blue). **(B)** The number of recoverin^+^ cells was diminished in ONA animals compared to the control group (p = 0.049). In ONA+C5I and ONA+C5II retinas the cell number remained unaltered compared to controls. **(C)** No differences in PKCα^+^ rod bipolar cells were noted within all groups. INL, inner nuclear layer. Values are mean ± SD. Scale bar = 20 µm. *p < 0.05.

The number of recoverin^+^ cells (n = 6–8/group) was significantly diminished in ONA retinas compared to controls (p = 0.049). However, no alterations were noted in both treatment groups in comparison to the control group (ONA+C5-I: p = 0.69; ONA+C5-II: p = 0.99, **Table 6**; **Figures 7A, B**).

Regarding PKCα^+^ rod bipolar cells (n = 6–8/group), cell counts in the ONA and the control group were comparable (p = 0.20). Also, no differences were found between both treatment groups and control retinas (both p = 0.99, **Table 6**; **Figure 7A, C**).

### Protection of Photoreceptors After Complement Inhibition

**Figure 8:**
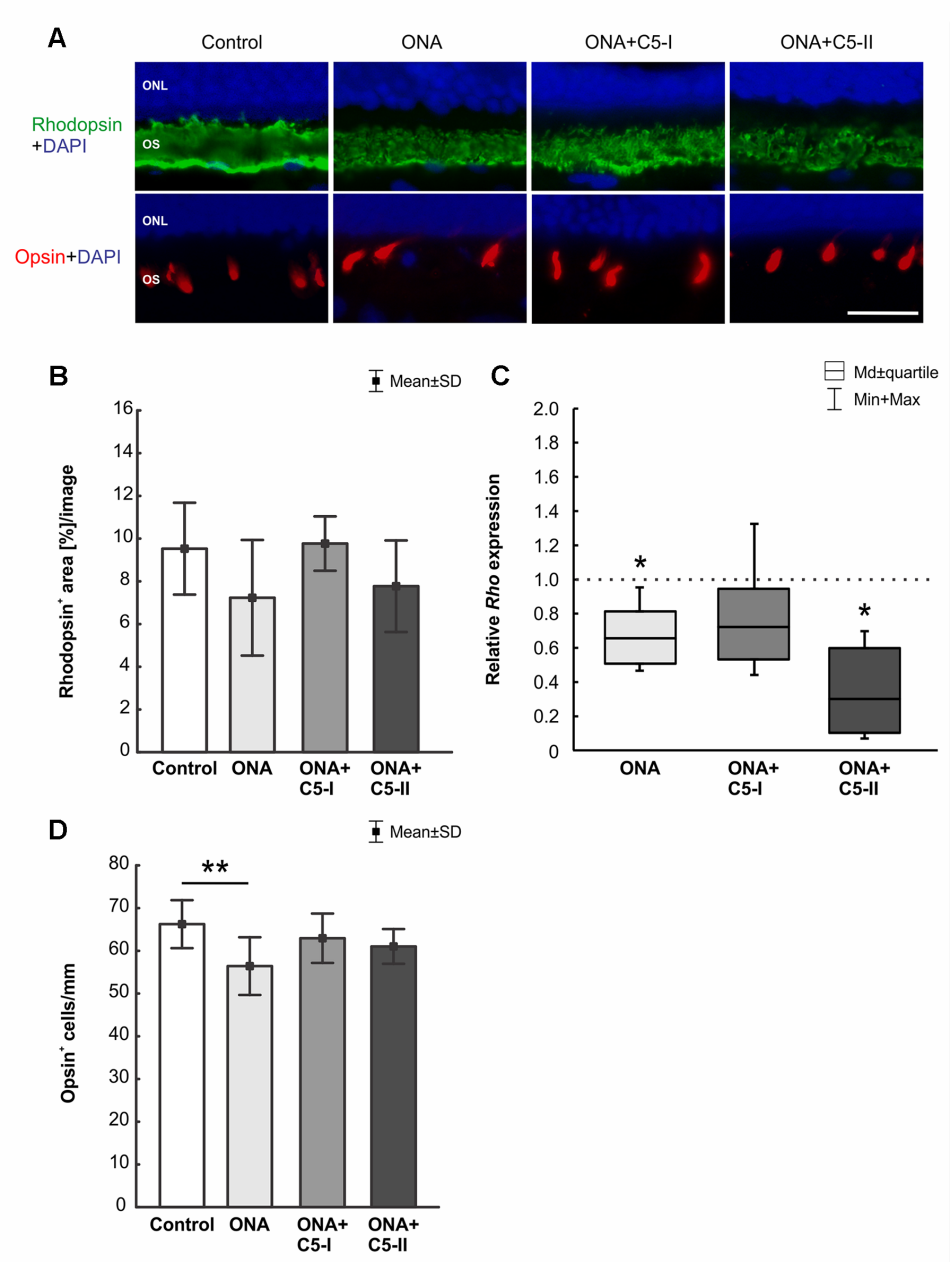
Protection of photoreceptors after treatment. **(A)** Rods were labeled with rhodopsin (green), L-cones were stained with opsin (red), and DAPI (blue) marked cell nuclei. **(B)** The rhodopsin^+^ area in the outer segments was similar in all groups. **(C)** However, a significant downregulation of *Rho* mRNA was observed in ONA and in ONA+C5-II retinas compared to controls (both: p = 0.014). **(D)** Less opsin^+^ cones were observed in ONA animals compared to control retinas (p = 0.009). No differences were observed in the treated groups in comparison to controls. Values for immunostaining are mean ± SD. Values for RT-qPCR are median± quartile ± maximum/minimum. The dotted line in C represents the relative expression level of the control group. ONL, outer nuclear layer; OS, outer segment. Scale bar = 20 μm. *p < 0.05, **p < 0.01.

The rhodopsin^+^ area was similar in all groups (n = 6–8/group). No difference could be detected between the stained area of the control, the ONA (p = 0.16), the ONA+C5-I (p = 0.99), and the ONA+C5-II group (p = 0.40; **Table 6**; **Figures 8A, B**). The mRNA expression level of *Rho* (n = 5/group) was not altered in ONA+C5-I animals compared to controls (0.72-fold expression; p = 0.10, **Figure 8C**). However, a significant downregulation of *Rho* mRNA was observed in ONA (0.66-fold expression; p = 0.014) and in ONA+C5-II retinas (0.302-fold expression; p = 0.014) compared to controls.

Counts of L-opsin^+^ cones (n = 6–8/group) revealed fewer cells were observed in ONA animals compared to control retinas (p = 0.009). While no differences were observed in the ONA+C5-I (p = 0.66) and ONA+C5-II retinas (p = 0.34) compared to controls (**Table 6**; **Figures 8A, D**).

## Discussion

Glaucoma is one of the most leading causes for blindness worldwide. However, the only treatable factor to date is lowering the IOP. Therefore, the aim of this work was to investigate, whether an intravitreal administration of an antibody against complement factor C5 can prevent the development and progression of the glaucomatous damage in the IOP-independent EAG model. Here, an immunization with ONA results in a complex systemic immune response, which then leads to apoptosis of RGCs as well as optic nerve degeneration (Laspas et al., 2011; Joachim et al., 2013; Noristani et al., 2016).

Since several years, the contribution of the immune system in glaucoma disease moved more and more into the focus in glaucoma research (Tezel and Wax, 2004; Grus et al., 2008; Wax, 2011). Especially, a dysregulation of the complement system seems to contribute to glaucoma damage. For example, significantly more C1q, C3, and MAC depositions were described in animal models with an elevated IOP (Kuehn et al., 2006; Jha et al., 2011; Becker et al., 2015). In these OHT studies, it could be demonstrated that a depletion of the complement system through the cobra venom factor reduces the RGC loss due to inhibition of the apoptotic pathways (Jha et al., 2011). Additionally, C5 deficient glaucomatous DBA/2J mice showed reduced neurodegeneration in comparison to control animals. Here, inhibition of complement activation is accompanied with reduced MAC deposition and RGC loss (Howell et al., 2013). Also, in the EAG model without IOP elevation, an activation of the complement system *via* the lectin pathway could be noted prior to cell death (Reinehr et al., 2016a; Reinehr et al., 2018b). In the study presented here, we therefore wanted to determine whether an inhibition of the complement system could preserve retinal degeneration after ONA immunization. In the complement cascade, the cleavage of C5 into C5a and C5b, and the following MAC formation, is a critical event. In an experimental autoimmune uveitis (EAU) model, intravitreal therapy with a monoclonal C5 antibody reduced infiltration and structural damage in the retina (Copland et al., 2010). Hence, we administered the same antibody also intravitreally, starting one day before ONA immunization and repeated this procedure every two weeks. The injection did not alter the IOP, which stayed within the normal range in all groups throughout the study. Previous studies using the EAG model also noted that the immunization did not increase the IOP (Laspas et al., 2011; Joachim et al., 2013; Casola et al., 2015). Furthermore, SD-OCT as well as H&E analyses showed that the retinal thickness was not affected and no inflammation or changes in the morphology occurred, as described before (Casola et al., 2015; Casola et al., 2016). Since comparable results were observed between SD-OCT and H&E examinations, in further studies the technique of OCT could be used as an *in vivo* tool to examine the retinal thickness overtime in animal studies.

One hallmark of glaucoma disease is a decline of RGCs (Weinreb and Khaw, 2004). RGCs are responsible for the transmission of visual stimuli from the retina to the brain *via* their axons (Yu et al., 2013; Mead and Tomarev, 2016). It is known that in the EAG model using ONA as an antigen, a RGC loss was noted 28 days after immunization (Laspas et al., 2011; Kuehn et al., 2016; Noristani et al., 2016). The results presented here also depicted RGC loss after ONA immunization, which could be preserved by administration of the C5 antibody. The C5 antibody was able to inhibit formation of the terminal MAC complex, which was previously shown in an EAU model (Copland et al., 2010). This MAC decrease likely inhibits apoptotic pathways. In our study, a downregulation of the *Bax*/*Bcl2* ratio was observed in the ONA+C5-I animals. It is known that depositions of MAC on the cell membrane induces apoptosis (Sato et al., 1999; Hughes et al., 2000). Shi et al. reported that MAC promoted apoptosis in their used cone photoreceptor cell line (Shi et al., 2015). In an OHT glaucoma model, the depletion of the complement system reduced apoptotic RGCs (Jha et al., 2011). Since an imbalance between intra- and extracellular ion concentrations is caused by MAC, they noted that a complement activation is correlated with high intracellular calcium concentration (Carney et al., 1990; Jha et al., 2011). Consequently, this free calcium can induce apoptosis (Wang et al., 1999; Huang et al., 2005). We therefore assume that through C5 inhibition, formation of MAC was decreased, resulting in a lower apoptosis rate and hence preservation of RGCs. Interestingly, we could not note any differences in cleaved caspase 3^+^ RGCs. The ONA group not only showed less RGCs, but also fewer apoptotic RGCs compared to control animals and both treatment groups. Previous studies revealed apoptotic mechanisms at early stages of retinal degeneration in this model, just before a loss of RGCs were noted (Joachim et al., 2014). 28 days after ONA immunization, only few apoptotic cells were still detectable (Noristani et al., 2016). We therefore assume that the expression of cleaved caspase^+^ cells peaks at earlier points in time and that the RGC degeneration already occurred in our study.

The complement inhibition had no downregulating effect on microglia cells or the pro-inflammatory cytokine IL-1β in the treatment groups. Even more activated microglia could be observed in the eyes treated with the C5 antibody. This might be due to the intravitreal injection itself. It is known that an injection alone can increase the number of microglia in rat retinae (Sobrado-Calvo et al., 2007; Kuehn et al., 2017). Furthermore, Di Pierdomenico et al. showed that repeated intravitreal injections, as also performed in our study, even more enhanced the microglia response in rat retinae. In addition, they observed substance-dependent microglia reactivity. For example, bevacizumab led to a higher microglia response than another anti-VEGF antibody. It was shown in another study that bevacizumab was phagocytosed by rat retinal microglia cells, therefore the authors assume similar effects in their study (Di Pierdomenico et al., 2016; Meyer et al., 2016). Due to these results it could be assumed that repeated injections or even the antibody itself led to an increase in microglia numbers in the retinae. In further studies, the number of intravitreal injections should be decreased to avoid those glia related side effects. Furthermore, a vehicle treated group should be included, to rule out that some of the observed effects were only based on the intravitreal injection itself. However, in case of complement inhibition, the intravitreal administration is a more useful therapeutic approach compared with a systemic one. Local injections can revert side effects, like infections, which will occur after a systemic suppression of the complement system (Morgan, 1995; Copland et al., 2010; Barnum, 2017).

The inhibition of the complement component C5 does not seem to influence microglia activity. In a mouse model for Alzheimer’s disease, a knock-out of C1q was accompanied by less microglia activation (Fonseca et al., 2004). The same group demonstrated that microglia are the main source of C1q in the brain and therefore probably also in the retina (Fonseca et al., 2017) Since the inhibition of the complement system in our study affected the cascade at a later stage, microglia are likely not affected. The higher microglia activity and an upregulation of *Il1b* observed in our study might explain why not all RGCs could be preserved through this treatment.

Similar results were noted for macroglia. The immunohistochemistry results revealed no group differences. However, a significant upregulation of *Gfap* was observed in ONA animals, with or without complement inhibition. It is known that in the EAG model, if at all, rather a modest macroglia response is noted (Noristani et al., 2016; Reinehr et al., 2016b).

All these results again underline the multifactorial character of glaucoma disease and the important role of multi-level therapies for glaucoma patients in the future.

Although the EAG model is well-established, little is known about the retinal function after immunization. We therefore performed ERG measurements 6 weeks after immunization in all groups. The a-wave amplitude was not really affected in the ONA group. However, the mean values tended to be lower compared to controls. Since the a-wave reflects the signal transmission from the photoreceptors, one would not assume any alterations in a glaucoma model. Nevertheless, in late stages, a degeneration of photoreceptor cells was reported *via* ERG in glaucoma patients (Nork et al., 2000; Velten et al., 2001). Additionally, in different OHT animal models, an impairment of photoreceptors was noted (Heiduschka et al., 2010; Ortin-Martinez et al., 2015; Reinehr et al., 2019). The mild differences of the a-wave amplitude in our EAG model were accompanied by a loss of L-opsin^+^ cells and a downregulation of *Rho* mRNA levels in the ONA group. However, no alterations were noted in the ONA+C5-I animals, assuming a protective effect of the complement inhibition also on photoreceptors. As mentioned before, we also noted a significant downregulation of the *Bax/Bcl2* mRNA levels in ONA+C5-I animals. Since we used the total retina for RNA extraction and cDNA synthesis, these results are not limited to the RGCs, but reflect the general situation in the retina including apoptosis of the photoreceptors.

Regarding the b-wave amplitudes, which reflect the response from the inner nuclear layers, a significant reduction was observed in the ONA animals compared to controls, but not in the ONA+C5-I animals. In several other studies, alterations regarding recoverin were noted. For example, OHT induction led to a diminished immunoreactivity of recoverin (Cuenca et al., 2010). Also in the βB1-CTGF mice, a model for POAG, fewer recoverin cells were observed (Reinehr et al., 2019). The functional loss of the b-wave amplitude was accompanied by fewer recoverin^+^ cone bipolar cells in ONA retinas compared to controls in the study presented here. In both treatment groups, the number of cone bipolar cells was not altered. These results lead to the presumption that a loss of inner nuclear cells after ONA immunization could be rescued through complement inhibition. Nonetheless, the results of the b-wave amplitudes of the ONA+C5-I groups also show that not all cells could be preserved, since there were no statistical differences between ONA+C5-I animals and the ONA group.

Interestingly, the amplitudes of the a- and b-wave of the ONA+C5-II group were significantly reduced in comparison to controls. We assume this is due to the opacification of the optical media of the eyes, which was probably caused by the higher C5 antibody dose. It is not clear why the higher dosage produced this alteration, but we assume that the higher volume injected or the higher osmolality in the vitreum led to an opacification. However, no systemic side effects were observed with both concentrations. Many studies showed a higher incidence of cataract in patients that had had an intravitreal injection of different drugs (Satarian et al., 2017; Koc et al., 2018). As discussed earlier, in future studies, fewer intravitreal injections with lower volumes should be performed to avoid unwanted lens opacifications.

## Conclusion

Several studies revealed an involvement of the immune system in glaucoma disease. Especially, deposits of complement system factors could be observed in human glaucomatous eyes as well as in animal models with high IOP. In the EAG model, where RGC loss occurs IOP-independently, complement activation was noted prior cell loss. Here, we could demonstrate that an intravitreal injection of a C5 antibody prevented retinal function and RGC loss *via* the inhibition of MAC formation. These results strengthen the hypothesis of a crucial role of the complement system in glaucoma.

## Data Availability Statement

All datasets generated for this study are included in the article/**Supplementary Material**.

## Ethics Statement

The animal study was reviewed and approved by Landesamt für Natur, Umwelt und Verbrauchsschutz Nordrhein-Westfalen, 84-02-04.2013.A291.

## Author Contributions

SR performed experiments, analyzed data, and wrote the manuscript. SG, CG, and MA performed experiments and analyzed data. GS analyzed data. MS performed experiments. HD revised the manuscript. SJ designed the study and revised the manuscript. All authors have read and approved the final manuscript.

## Funding

This work was supported by the Deutsche Forschungsgemeinschaft (DFG, grant JO-886/1-3). SR was supported by the Ernst and Berta Grimmke foundation (Germany).

## Conflict of Interest

The authors declare that the research was conducted in the absence of any commercial or financial relationships that could be construed as a potential conflict of interest.

## Supplementary Material

Supplementary Figure 1 **(A)** The monoclonal antibody against C5 was administered before and every two weeks after ONA immunization. At 4 weeks, animals received a booster immunization. IOP was measured weekly. After 6 weeks, OCT, ERG as well as histological, immunohistological, and RT-qPCR analyses were performed. **(B)** Summary of markers, which were used in immunohistology and/or RT-qPCR experiments.
