# Supplementary figures and images for: Intravitreal Therapy Against the Complement Factor C5 Prevents Retinal Degeneration in an Experimental Autoimmune Glaucoma Model
Source: Front Pharmacol. 2019 Dec 2;10:1381. doi: 10.3389/fphar.2019.01381 (PMC6901014; doi:10.3389/fphar.2019.01381)

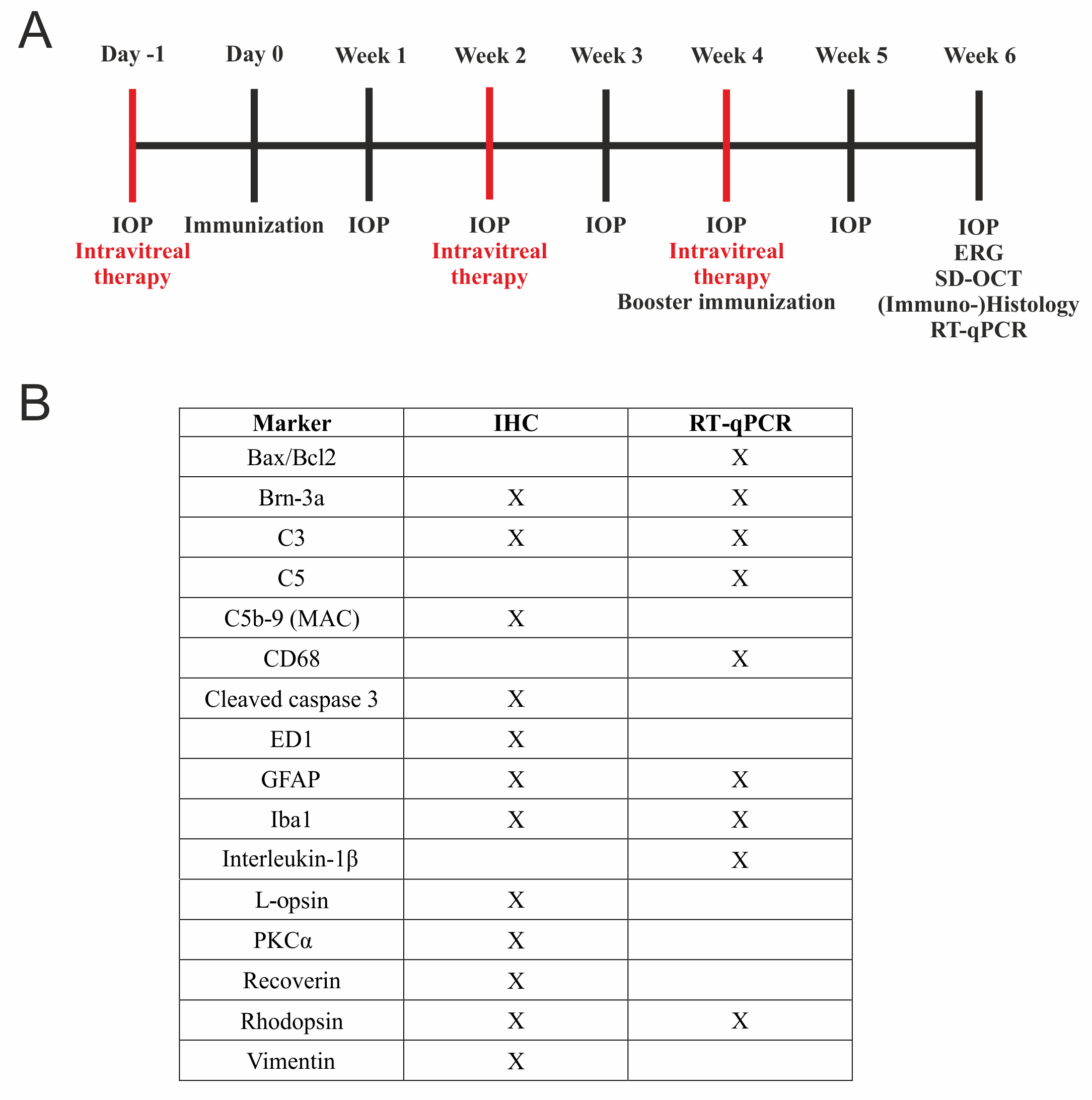

Supplement: Supplementary Figure 1 — (A) The monoclonal antibody against C5 was administered before and every two weeks after ONA immunization. At 4 weeks, animals received a booster immunization. IOP was measured weekly. After 6 weeks, OCT, ERG as well as histological, immunohistological, and RT-qPCR analyses were performed. (B) Summary of markers, which were used in immunohistology and/or RT-qPCR experiments. [file Image_1.tif]
